# A CRISPR-based method for testing the essentiality of a gene

**DOI:** 10.1038/s41598-020-71690-8

**Published:** 2020-09-08

**Authors:** Yan You, Sharmila G. Ramachandra, Tian Jin

**Affiliations:** Chemotaxis Signal Section, Laboratory of Immunogenetics, National Institute of Allergy and Infectious Diseases, National Institutes of Health, Rockville, MD USA

**Keywords:** Biological techniques, Cell biology

## Abstract

The CRISPR/Cas9 system is a powerful method of editing genes by randomly introducing errors into the target sites. Here, we describe a CRISPR-based test for gene essentiality (CRISPR-E test) that allows the identification of essential genes. Specifically, we use sgRNA-mediated CRISPR/Cas9 to target the open reading frame of a gene in the genome and analyze the in-frame (3n) and frameshift (3n + 1 and 3n + 2) mutations in the targeted region of the gene in surviving cells. If the gene is non-essential, the cells would carry both in-frame (3n) and frameshift (3n + 1 and 3n + 2) mutations. In contrast, the cells would carry only in-frame (3n) mutations if the targeted gene is essential, and this selective elimination of frameshift (3n + 1 and 3n + 2) mutations of the gene indicate its essentiality. As a proof of concept, we have used this CRISPR-E test in the model organism *Dictyostelium discoideum* to demonstrate that Dync1li1 is an essential gene while KIF1A and fAR1 are not. We further propose a simple method for quantifying the essentiality of a gene using the CRISPR-E test.

One of the most fundamental tasks of genetics is to identify genes that are essential for cellular or organismal viability^1^. Current molecular genetic approaches, such as transposon mutagenesis, gene trapping, homologous recombination^2^ and the more recently developed CRISPR gene editing^3–7^, identify essential genes by first inactivating them and then determining if the cells or organisms carrying these mutations are inviable. Because these approaches use negative selection, there is no guarantee and often no proof that the gene of interest has been disrupted or deleted (inactivated) in the dead cells. In the social amoeba *Dictyostelium discoideum*, a homologous-recombination method has been commonly used to knock out genes^2^. However, we found that it is impossible to knock out some genes, and in those cases, one cannot conclude with certainty that these genes are essential due to the lack of evidence that the desired recombination events have occurred. To overcome this problem, we developed a simple CRISPR-based essentiality test (named the CRISPR-E test) to determine whether a gene is essential by targeting a gene of interest followed by analyzing mutations within the gene in surviving cells.

To evaluate essentiality of gene X using the CRISPER-E test (Fig. 1), we express in cells the Cas9 protein and a sgRNA with a protospacer-adjacent motif (PAM) matching a location within gene X’s open-reading frame. Cas9 cuts DNA at the location to cause a double-strand break (DSB), and the DNA-repairing machinery fixes the break by non-homologous end joining (NHEJ), which ligates the ends and often introduces errors, including the insertion or deletion of a few nucleotides^8–11^. If gene X is non-essential, surviving cells would carry, beside wild-type (WT) alleles, various mutant alleles, including both frameshift mutations (3n + 1 and 3n + 2, n = …− 3, − 2, − 1, 0, 1, 2, 3…) that inactivate gene X and in-frame mutations (3n) that possibly retain the function of gene X. However, if gene X is essential, the cells would carry only in-frame mutations (3n) but no frameshift mutations (3n + 1 and 3n + 2). The presence of in-frame mutations proves that CRISPER/Cas9 has cut gene X, and that the DNA-repairing machinery has fixed the ends by NHEJ. This selective elimination of frameshift mutations from living cells, a natural selection process, should indicate the essentiality of the gene.

**Figure 1:**
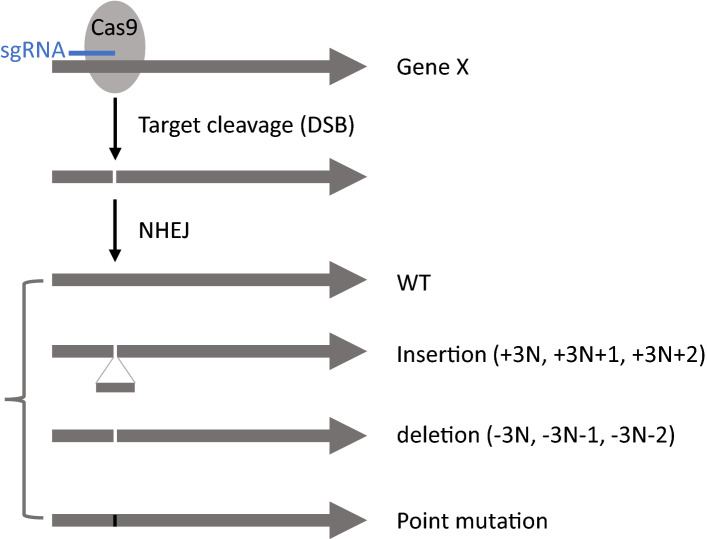
Mutational profiles of gene X generated by CRISPR/Cas9. A sgRNA matches a location and guides Cas9 to achieve a targeted cleavage at the location to cause double-strand breaks (DSBs), followed by non-homologous end joining (NHEJ). After this sgRNA/Cas9-mediated gene editing, the DNA sequence of gene X should include WT, deletion, insertion, and point mutations. WT alleles are considered as alleles that have not been modified by CRISPR/Cas9 due to the lack of proof of gene editing, and other mutations are regarded as the products of gene editing. Edited genes include WT or mutant alleles with insertion, deletion and point mutations. Among them, frameshift mutations are 3n + 1, 3n + 2, and in-frame mutations are 3n (n = …− 3, − 2, − 1, 0, 1, 2, 3…). The positions of cutting sites on the codon (1st, 2nd or 3rd) do not influence the outcome of generating frameshift mutations: Regardless of the position, 3 N + 1 or 3 N + 2 will generate frameshift mutations, but 3 N will mostly generate in-frame mutations with amino acid insertions or deletions and one or two amino acid substations dependent on the cutting sites on the codon. There is a small chance that a 3 N mutation with a created stop code at a cutting site could also knockout the function of the gene. To confirm the in-frame or frameshift mutations, DNA sequence will be translated into protein sequence.

A CRISPR/Cas9 system has been developed to edit genes in *D. discoideum*^12^. To demonstrate the effectiveness of sgRNA-mediated CRISPR/Cas9 method, we first edited two genes, grlB (DDB_G0271686) and grlC (DDB_G0282461)^13^, by designing sgRNAs that target at the coding region of a gene (Fig. 2). Using web-based tools, E-CRISP or Cas-Designer, we obtained a list of potential sequences adjacent to PAM with several SAE scores, which are calculated based on sequence homology and the number and positions of mismatches relative to the sgRNA sequence. To minimize off-target editing, we chose sgRNAs with the highest on-target efficiency. We selected two sgRNAs for each of grlB (Fig. 2a and S. Figure 1a and S. Figure 2a) and grlC gene (Fig. 2b, S. Figure 3a and S. Figure 4a) and inserted a gRNA scaffold-sgRNA sequence into an expressing cassette and generated a vector expressing Cas9, GFP and G418-resistance cassette using pTM1285 as the parental plasmid^12^. To edit the grlB gene, a plasmid encoding sgRNA1 for grlB was transformed into wild-type (AX2) cells, and transformants were selected in culture medium and then plated on SM agar with *Klebsiella aerogenes*. A total of 12 clones, grlB-B1-B12, were obtained (S. Figure 1a). To sequence the target region edited by sgRNA/Cas9 in individual clones, we amplified genomic DNA of the coding region surrounding sgRNA1 from each of the 12 clones using PCR with designed primers (S. Figure 1a) and sequenced the PCR products from each clone (S. Figure 1b). Sequencing chromatogram showed that the grlB gene was edited by sgRNA1 in 6 clones and generated both in-frame mutants such as grlB_B1(+ 12 bp) and grlB_B10 (+ 3 bp) as well as frameshift mutants such as grlB_B2 (+ 4 bp), grlB_B4 (− 4 bp), grlB_B9 (− 22 bp), and grlB_B12 (+ 7 bp, 1 s) (Fig. 2a and S. Figure 1b), while the sgRNA1-mediated editing failed in 6 clones (grlB_B3, grlB_B5, grlB_B6, grlB_B7, and grlB_B11) as we did not get clear sequencing results (S. Figure 1b). The in-frame (2 out of 6, 33%) and frameshift mutations (3n + 1: 2 out of 6, 33%; 3n + 2: 2 out of 6, 33%) were confirmed by an analysis of the protein sequences translated from the DNA sequences (Fig. 2a). To further test the method, we also transformed a plasmid encoding sgRNA2 for grlB into wild-type cells and randomly selected 12 clones (S. Figure 2a). Genomic DNA of the coding region around sgRNA2 was amplified by PCR and then sequenced (S. Figure 2a). Sequencing showed that each of the 12 clones carried wild-type sequence in the coding region around sgRNA2, demonstrating that sgRNA2-mediated edition did not work.

**Figure 2:**
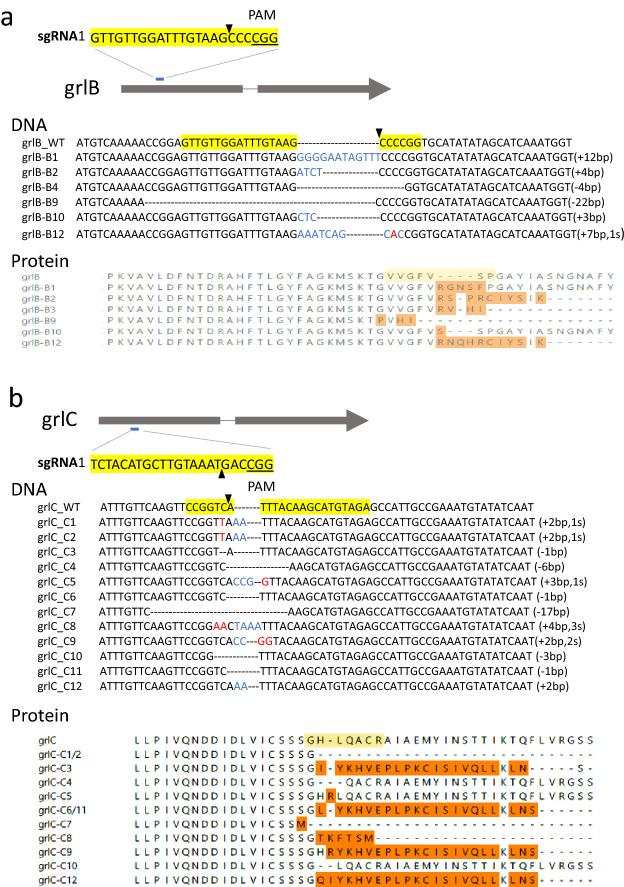
sgRNA-mediated gene edition (**a**) A schematic view of sgRNA1 targeting the grlB gene. The yellow-shaded sequence of sgRNA1 targets on the open reading frame of grlB. Black triangles indicate the expected cutting sites. Under the label “DNA”, we show the coding strand DNA from the sequencing results of the target regions from wild-type cells (grlB_WT) and 6 individual clones (grlB_B1, grlB_B2, grlB_B4, grlB_B9, grlB_B10, and grlB_B12). Red letter indicates an insertion of a nucleotide, a dash shows a deletion of a nucleotide, and blue letter shows substitution of a nucleotide. The in-frame mutations (3n) are 33% (2 out of 6), and frameshift mutations (3n + 1 and 3n + 2) are 33% (2 out of 6) and 33% (2 out of 6), respectively. Under the label “Protein”, we show the translated protein sequences of WT and grlB_B1, grlB_B2, grlB_B4, grlB_B9, grlB_B10, and grlB_B12. Note that grlB_B2, grlB_B3, grlB_B9 and grlB_B12 are frameshift mutations. The yellow-shaded sequence indicates the target site of sgRNA. (**b**) A schematic view of sgRNA1 targeting grlC gene. The yellow-shaded sequence of sgRNA1 targets on the open reading frame of grlC. Black triangles indicate the expected cutting sites. Under the label “DNA”, we show the coding strand DNA from the sequencing results of the target regions from wild-type cells (grlC_WT) and 12 individual clones (grlC-C1-grlC_C12). The in-frame mutations (3n) are 30% (3 out of 10), and frameshift mutations (3n + 1 and 3n + 2) are 20% (2 out of 10) and 50% (5 out of 10), respectively. Red letter indicates an insertion of a nucleotide, a dash shows a deletion of a nucleotide, and blue letter shows substitution of a nucleotide. Under the label “Protein”, we show the translated protein sequences of WT, grlC_C1-grlC_C12.

**Figure 3:**
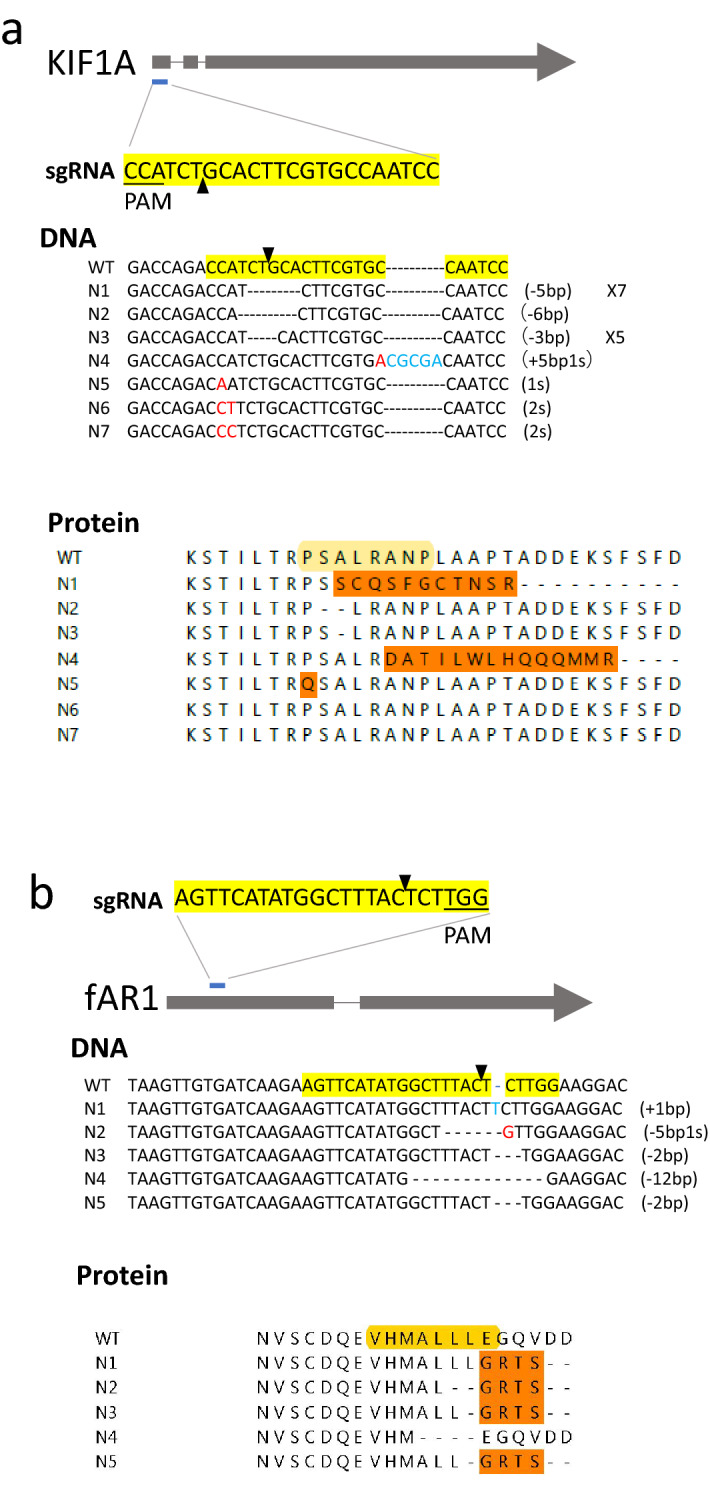
sgRNA-mediated edition of the KIF1A and fAR1 genes. (**a**) A schematic view of sgRNA targeting KIF1A. The yellow-shaded sequence of sgRNA targets on the open reading frame of KIF1A. Black triangles indicate the expected cutting sites. Under the label “DNA”, we show the non-coding strand DNA from the sequencing results of the target regions from 19 individual clones (2WT, 7N1, N2, 5N3, N4, N5, N6, and N7). Red letter indicates a substitution of a nucleotide, a dash shows a deletion of a nucleotide, and blue letter shows an insertion of a nucleotide. Under the label “Protein”, we show the translated protein sequences of WT and N1-N9. N1, N2, N3 and N6 are frameshift mutations. The yellow-shaded sequence indicates the target site of sgRNA. (**b**) A schematic view of sgRNA targeting fAR1. The yellow-shaded sequence of sgRNA targets on the open reading frame of fAR1. Black triangles indicate the expected cutting sites. Under the label “DNA”, we show the coding strand DNA from the sequencing results of the target regions from five individual clones (N1-N5). Under the label “Protein”, we show the translated protein sequences of WT and N1-N5. N1-N4 and N5 are frameshift mutations.

**Figure 4:**
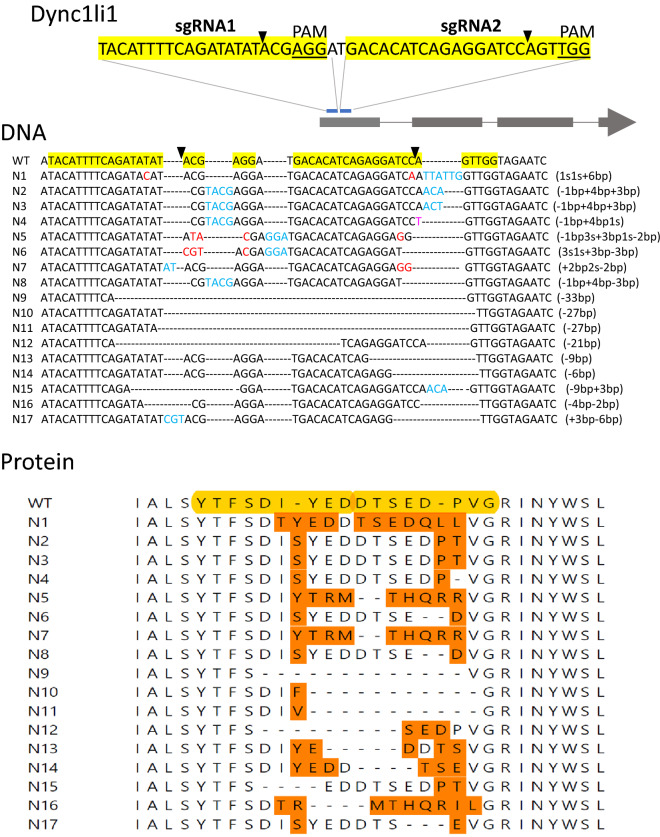
The CRISPR-E method shows that Dynclli1 is essential. A schematic view of sgRNA1 and sgRNA2 targeting Dync1li1 gene. The yellow-shaded sequences show sgRNA1 and sgRNA2 sequences targeting the open reading frame of Dynclli1. Black triangles indicate the expected cutting sites. Under the label “DNA”, we show the coding strand DNA from sequencing results of the target regions of 21 individual clones, which includes 3WT, N1-14, 2N15, N16 and N17. Red letter indicates a substation of a nucleotide, a dash shows a deletion of a nucleotide, and blue letter shows an insertion of a nucleotide. Under the label “Protein”, we show translated protein sequences of WT and N1-N17. Yellow-shaded sequences show the regions targeted by sgRNA1 and sgRNA2. All mutant alleles contain in-frame (3n) mutations.

To edit grlC, we transformed a plasmid expressing either sgRNA1 (Fig. 2b and S. Figure 3a) or sgRNA2 (S. Figure 4a) and randomly picked 12 clones for sgRNA1-mediated edition (S. Figure 3a) or sgRNA2-mediated edition. Subsequently, we amplified genomic DNA using PCR and sequenced the coding regions surrounding either sgRNA1 (Fig. 2b and S. Figure 3a and 3b) or sgRNA2 (S. Figure 4a and 4b). Sequencing chromatogram showed that all 12 clones were edited by sgRNA1 (Fig. 2b). Among them, grlC_C1/grlC_C2 and grlC_C6/grlC_C11 were two pairs of identical sequences, indicating that a pair of mutants were likely the two progenies of one gene-editing event, and therefore, grlC-C1/grlC_C2 and grlC_C6/grlC_C11 were considered as two individual clones. Among the 12 sequenced clones, we found 10 independent gene-editing events that modified sequences around sgRNA1 (Fig. 2b), including 3 in-frame mutations (grlC_C4 − 6 bp; grlC_C5 + 3 bp, 1 s; grlC_C10 − 3 bp in Fig. 2f) and 7 frameshift mutations (grlC_C1/C2 + 2 bp,1 s; grlC_C3 − 1 bp; grlC_C6/C11 − 1 bp; grlC_C7 − 17 bp; grlC_C8 + 4 bp,3 s; grlC_C9 + 2 bp,2 s; grlC_C12 + 2 bp in Fig. 2f). The in-frame (3 out of 10, 30%) and frameshift (3n + 1: 2 out of 10, 20%; 3n + 2: 5 out of 10, 50%) mutations were confirmed by protein sequences translated from the DNA sequences (Fig. 2b). Gene edition of grlC by sgRNA2 did not work (S. Figure 4). Sequencing data from the 12 clones showed that 8 clones carried wild-type sequence of grlC gene and 4 clones did not display clear sequences (S. Figure 4b). Taken together, our results indicated that sgRNA-mediated gene edition successfully generated in-frame and frameshift mutations when the method worked properly, and when all clones showed either wild-type sequence or no clear sequencing results, we knew that the sgRNA-mediated gene edition failed.

To experimentally determine the effectiveness of generating loss-of-function mutations, we examined three genes, KIF1A (encoding kinesin-3), fAR1 (encoding the folic-acid receptor) and Dync1li1 (encoding dynein light intermedium chain) in *D. discoideum*. KIF1A and fAR1 have been previously knocked-out by homologous recombination and thus are known to be non-essential for cell proliferation but the mutants displayed clear phenotypes^14,15^, while Dync1li1 has not been knocked-out and has been suggested to be essential for the proliferation of *D. discoideum* cells^16^. To ensure the generation of a frameshift mutation causing a loss-of-function of the gene, we selected sgRNAs targeting at the 3′ coding region of the first exon, which is not close to an intron. Using this strategy, we transformed cells with a vector expressing Cas9 and designed sgRNAs targeting KIF1A (Fig. 3a), fAR1 (Fig. 3b) and Dync1li1 (Fig. 4 and S. Figure 5), respectively. After the transformation of a sgRNA/Cas9-containing vector, the cells were plated on the SM agar with a bacterial lawn where they would grow as individual clones. We then randomly picked independent clones, amplified the target regions by PCR from each of the clones and sequenced the PCR products. Among 19 sequenced clones in which the sgRNA target was on KIF1A, two were WT, seven contain a 5-bp deletion at different positions, one contains a 6-bp deletion, five contain a 3-bp deletion, and, one contains a 5-bp insertion and a 1-bp substitution, and three contain point-mutations (Fig. 3a, DNA). There were eight frameshift mutations (3N1, 2N2, 2N3 and 1N6 in Fig. 3a, Protein), and each of the frameshift mutants displayed the expected phenotype of KIF1A-null cells, indicating that the frameshift mutations inactivated KIF1A function. Thus, the efficiency of on-target cutting/editing by sgRNA/Cas9 on KIF1A is about 89.5% (17 out of 19) and the efficiency of gene inactivation (frameshift mutations) is about 42% (8 out of 19). Among five sequenced fAR1 clones (Fig. 3b), there were one in-frame mutation and four frameshift mutations, each of which showed the expected fAR1-null phenotype. We rescued the phenotype of N1 and N2 by expressing fAR1-GFP (data not shown). Our results indicated that sgRNA-mediated fAR1 gene edition generated the loss-of-function mutations, and that the efficiency of on-targeting cutting/editing on fAR1 is about 100% and the efficiency of gene inactivation (frameshift mutations) is 80% (4 out of 5).

The result from the Dync1li1 gene is drastically different (S. Figure 5 and Fig. 4), and our CRISPR-E test indicates that Dync1li1 is an essential gene for cell growth as predicted. Specifically, we sequenced eight clones of Dync1li1 targeted by sgRNA1 and ten clones targeted by sgRNA2 (S. Fig. 5) and found only wild-type (WT) alleles or mutant alleles with in-frame mutations (sgRNA1: four WT, two point mutations, one 3-bp insertion and one 21 bp deletion; sgRNA2: three WT, two point mutations, one 6-bp deletion, one 9-bp deletion, and three 3-bp deletions). While on-targeting cutting/editing efficiency were 50% and 70% respectively, the efficiency of gene inactivation was 0. Assuming that CRISPR-mediated gene editing randomly generates 3n, 3n + 1, and 3n + 2 mutations, the probability of obtaining four and seven in-frame (3n) mutations by sgRNA1 and sgRNA2 are P1 = (1/3)^4^ = 0.005 and P2 = (1/3)^7^ = 0.00018, respectively. The absolute dominance of these low probability events indicates that frameshift (3n + 1 and 3n + 2) mutations have been eliminated by natural selection during cell proliferation. To further increase the efficiency of cutting/editing on Dync1li1, we co-transformed vectors of sgRNA1/Cas9 and sgRNA2/Cas9 into cells and sequenced 21 individual clones. There are three WT and eighteen mutations, and the gene had been heavily edited on the region targeted by sgRNA1 and sgRNA2 with an 85% on-target efficiency (Fig. 4 DNA). However, each mutation was 3n resulting in a Dync1li1 protein with deletions, insertions or (and) substitutions of a few amino acids in the targeted region (Fig. 4 Protein), and not a single frameshift mutation was detected (Fig. 4 DNA). We interpret this result as that frameshift mutations were eliminated because inactivating Dync1li1 is lethal to cell proliferation.

The CRISPR-E test may provide a quantitative measurement of the essentiality of a gene. The efficiency of CRISPR-based gene cutting/editing is high and can be measured experimentally. Assuming that mutations generated from NHEJ are random, we can develop mathematical models/computing programs to calculate the probabilities of mutations. Experimentally measured frequency of gene inactivation (frameshift mutations of a gene) divided by the theoretical frequency gives rise to a number E, which provides a measurement of a gene’s essentiality. E > 1 means that inactivating the gene is beneficial for cell’s survival, E = 1 means that inactivating the gene has no effect on survival, 1 > E > 0 means that inactivating the gene has a negative effect on survival, and E = 0 means that the gene is essential. CRISPR-E test is a simple method that can be applied to measuring quantitatively the essentiality of every gene in haploid or diploid cells of all organisms. If a gene is not essential for cell growth and proliferation, its essentiality can be further examined during other biological processes, such as cell migration, cell differentiation, and development of multicellular organisms. However, some studies indicated that mutations generated by a sgRNA may be biased^17–19^. Thus, while this method should work on essential genes it may not be reliably used for quantifying the essentiality of a non-essential gene. Future research efforts are required to quantitatively measure the essentiality of genes of interest in various organisms and to screen for those that are critical for various biological processes, by using this simple CRISPR-E method in different genetic and environmental contexts.

## Methods

### Strains, cell culture, plasmid construction and PCRs

*Dictyostelium discoideum* strains AX2 (wild type) and their transformants were incubated at 22 °C on culture dishes or in shaking culture in HL5 medium or on SM agar with *Klebsiella aerogenes*. Transformants were selected in HL5 medium with 20 µg/ml of G418. Using pTM1285 as the parental plasmid^12^, we inserted a gRNA scaffold-sgRNA sequence into the sgRNA-expressing cassettes and generated vectors expressing Cas9, GFP, G418-resistance cassette and sgRNA targeting KIF1A, fAR1 or Dync1li1. Each of the plasmids was transformed into AX2 (wild-type) cells. To sequence the target regions edited by sgRNA/Cas9 in individual clones, we amplified genomic DNA using PCR with the following primers: KIF1AF (5′-GAAGAGCAAGGTAAAAAGG-3′), KIF1AR (5′-CCTTTTACCAGAACCAGTTTG-3′), and the PCR product is 400 bp for WT; for fAR1, fAR1F (5′-ACGACCCATTGTATTAT-3′), fAR1R (5′- GACTTTGAGTACA AATATCG-3′), and the PCR product is 530 bp for WT; for Dync1li1, Dync1li1(5′-AAGAAGATATTTGGGGTC-3′), Dync1li1R (5′-GGTTGTGAAAAATCTAAAG-3′), and the PCR product is 337 bp for WT; grlBF (5′ AGTGGTCAAACTGAAATGGG 3′), grlBR (5′TCAATGTTTTCTTCTGGTTGT 3′) for grlB sgRNA 1 and the PCR product is 1901 bp for WT; grlB8F (5′TGTATCCATGGGTAGCTGGT3′), grlB8R (5′GCGGCACCTTGAGTTATCAT3′) for grlB sgRNA2 and the PCR product 448 bp for WT; grlC7F (5′TCAAGGTAGAATTGAAGTTGCCA3′), grlC7R (5′TCTTGCGCCAACCCAAAATG3′) for grlC sgRNA1 and the PCR product is 400 bp for WT; grlCF (5′GGTAGAATTGAAGTTGCCAAAGGT3′), grlCR.

(5′GCTAGTACAGCACCAGCTACA3′) for grlC sgRNA2 and the PCR product is 1988 bp for WT.

## Supplementary information

Supplementary Information.
