# Supplementary material for: A CRISPR-based method for testing the essentiality of a gene
Source: Sci Rep. 2020 Sep 8;10:14779. doi: 10.1038/s41598-020-71690-8 (PMC7478968; doi:10.1038/s41598-020-71690-8)
Supplement: Supplementary file 1 — Supplementary Information. [file 41598_2020_71690_MOESM1_ESM.pdf]

Supplementary information

A CRISPR-based method for testing the essentiality of a gene

Yan You#, Sharmila G. Ramachandra#, Tian Jin*

Chemotaxis Signal Section, Laboratory of Immunogenetics, National Institute of Allergy and Infectious Diseases, National Institutes of Health, Rockville, MD.
#Authors contribute equally
*tjin@niaid.nih.gov

Supplementary Fig. 1. a. The coding sequence of grlB and the locations of sgRNA1 (brown box) with PAM sequence, forward primer (green) and reverse primer (red). Picture of SM KA plates with individual clones and PCR products from these selected clones. **b**. Chromatogram of sequence of grlB from the clones of sgRNA1-mediated gene edition.

Supplementary Fig. 2. **a**. The coding sequence of grlB and the locations of sgRNA2 (brown box) with PAM sequence, forward primer (green) and reverse primer (red). **b**. Chromatogram of sequence of grlB from the clones of sgRNA2-meidated gene edition.

Supplementary Fig. 3. a. The coding sequence of grlC and the locations of sgRNA1 (brown box) with PAM sequence, forward primer (green) and reverse primer (red). Picture of SM KA plates with individual clones and PCR products from these selected clones. **b**. Chromatogram of sequence of grlC from the clones of sgRNA1-mediated gene edition.

Supplementary Fig. 4. **a**. The coding sequence of grlC and the locations of sgRNA2 (brown box) with PAM sequence, forward primer (green) and reverse primer (red). **b**. Chromatogram of sequence of grlC from the clones of sgRNA2-mediated gene edition.

Supplementary Fig. 5. **a**. A schematic view of sgRNA1 and sgRNA2 targeting Dync1li1 gene. The yellow-shaded sequences show sgRNA1 or sgRNA2 sequences targeting the open reading frame of Dynclil. **b**. Dync1li1 gene edited by sgRNA1. Under the label "DNA", we show the sequencing results of the target regions of eight individual clones, which includes 4WT, N1-N4. Red letter indicates a substation of a nucleotide, a dash shows a deletion of a nucleotide, and blue letter shows an insertion of a nucleotide. Under the label "Protein", we show the translated protein sequences of WT and N1-N4. Yellow-shaded sequences show the regions targeted by sgRNA1. **c.** Dync1li1 gene edited by sgRNA2. Under the label "DNA", we show the sequencing results of the target regions of ten individual clones, which includes three WT, N1-N7. Red letter indicates a substation of a nucleotide, a dash shows a deletion of a nucleotide, and blue letter shows an insertion of a nucleotide. Under the label "Protein", we show the translated protein sequences of WT and N1-N7. Yellow-shaded sequences show the regions targeted by sgRNA2. All mutant alleles contain in-frame (3n) mutations.

S. Fig 1 a

grlB sgRNA1

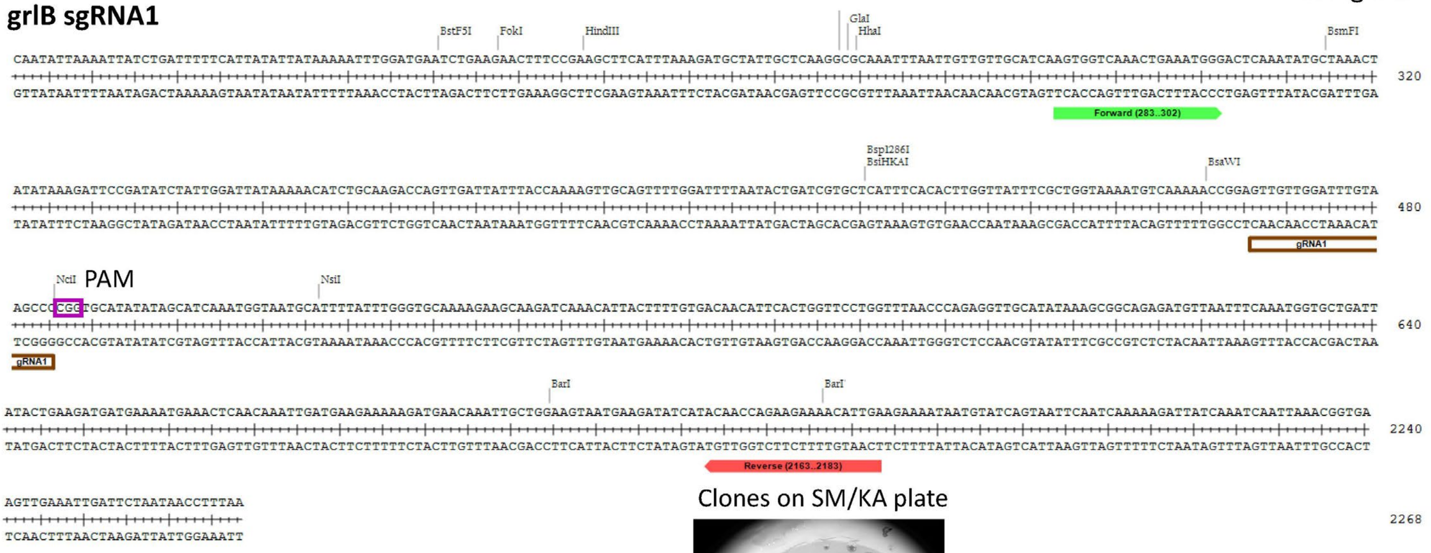

Clones on SM/KA plate

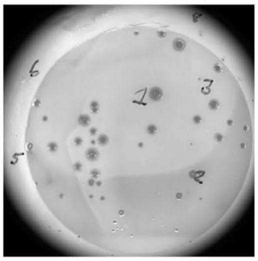

PCR porducts

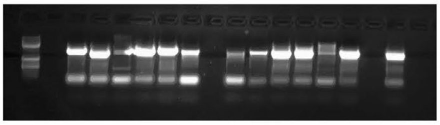

forward primer was used for sequencing

Primers

F: 5’-AGTGGTCAAACTGAAATGGG 3’ (283bp- 302bp)

R: 5’-TCAATGTTTTCTTCTGGTTGT 3’ (2163bp- 2183bp

Product size: 1901bp

6/26/2020

S. Fig. 1b

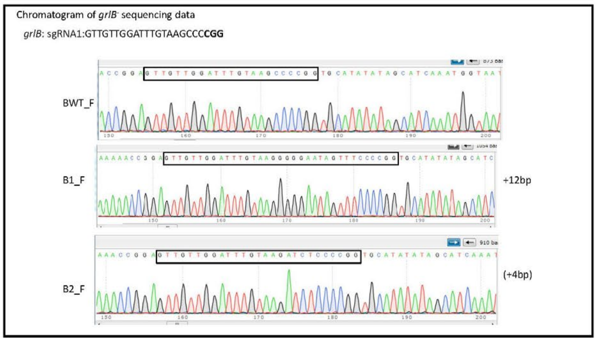

1

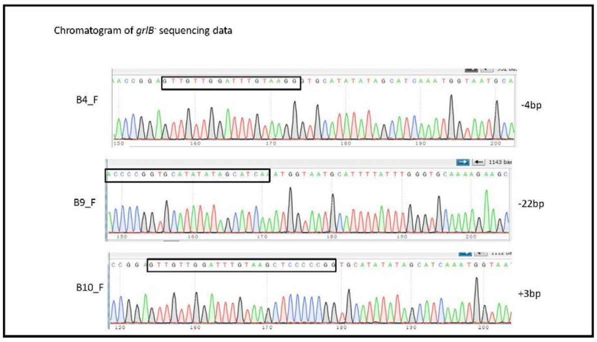

2

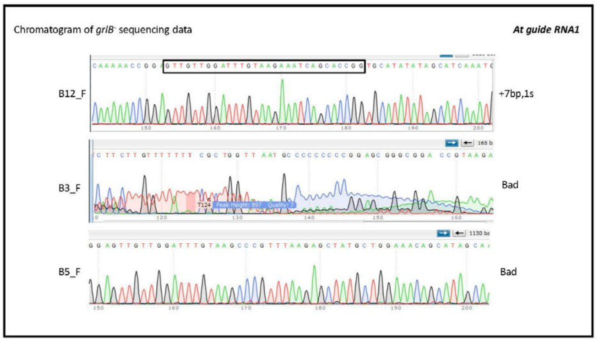

3

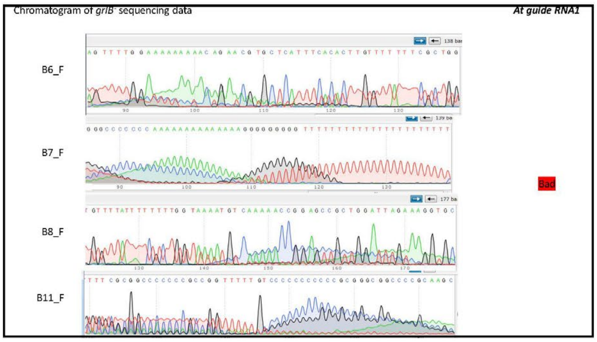

4

grlB sgRNA2

S. Fig. 2a

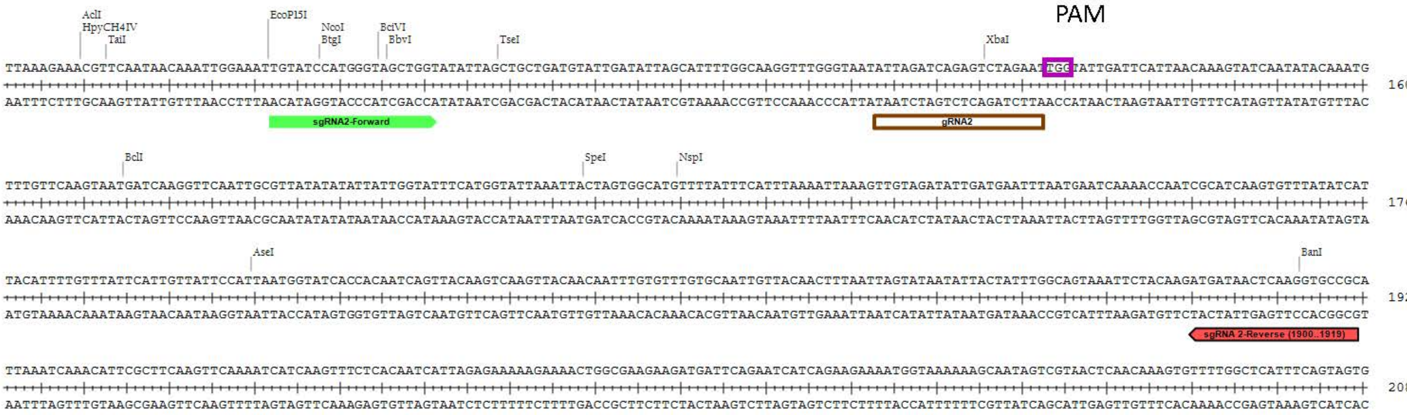

For guide2
F: TGTATCCATGGGTAGCTGGT (1472bp- 1491bp)
R: GCGGCACCTTGAGTTATCAT (1900bp- 1919bp)
Product size:448bp

forward primer was used for sequencing

6/27/2020

S. Fig. 2b

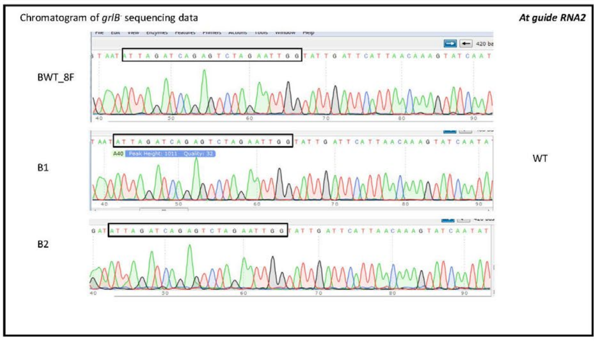

1

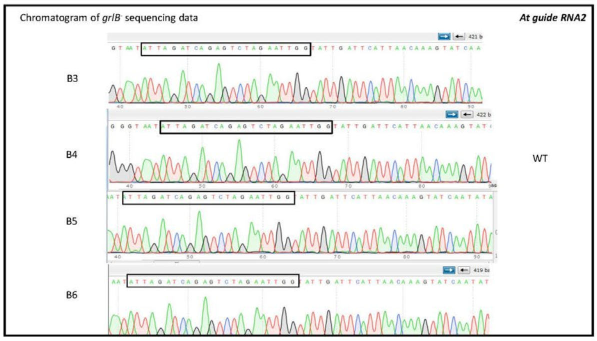

2

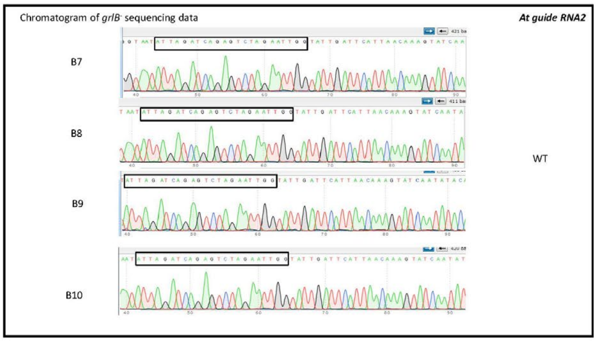

3

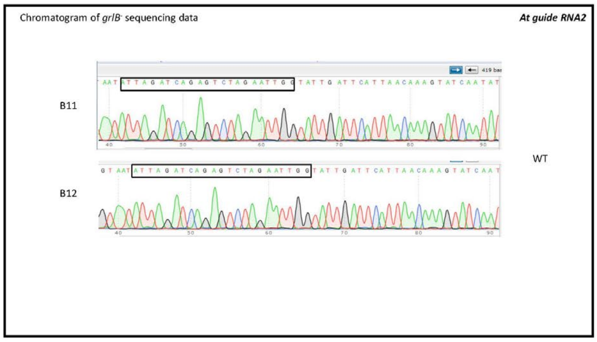

4

S. Fig. 3a

grlC sgRNA1

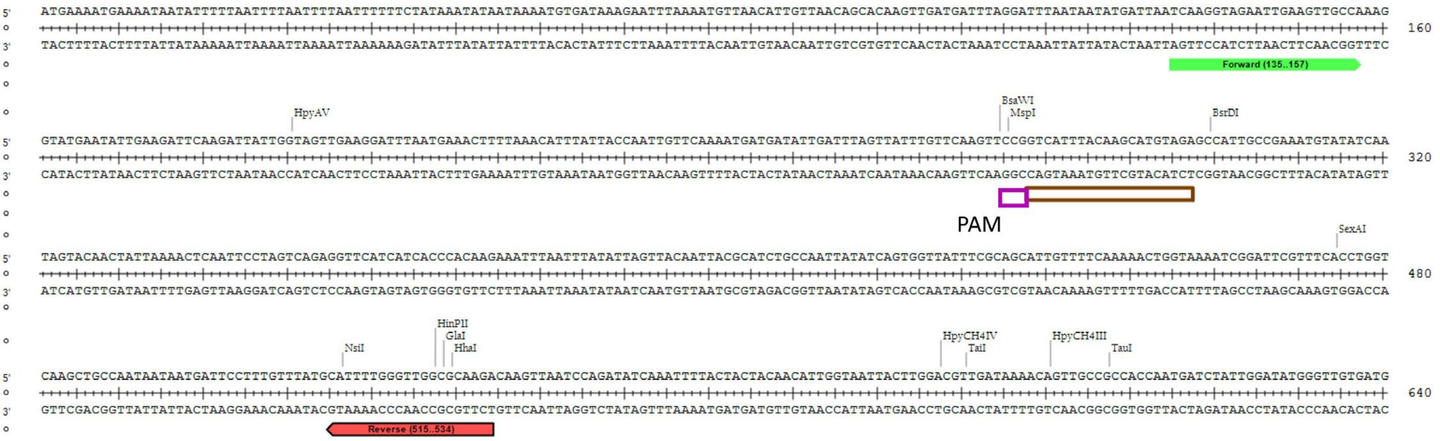

Reversed primer was used for sequencing

Primers

guide1

F: 5’-TCAAGGTAGAATTGAAGTTGCCA-3’ (135bp -157bp)

R:5’-TCTTGCGCCAACCCAAAATG-3’ (515bp- 534bp)

Product size:400bp

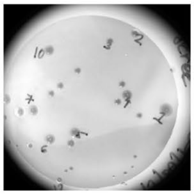

On KA

grlC⁻ screening

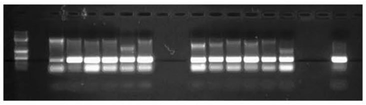

PCR products

6/27/2020

S. Fig.3b

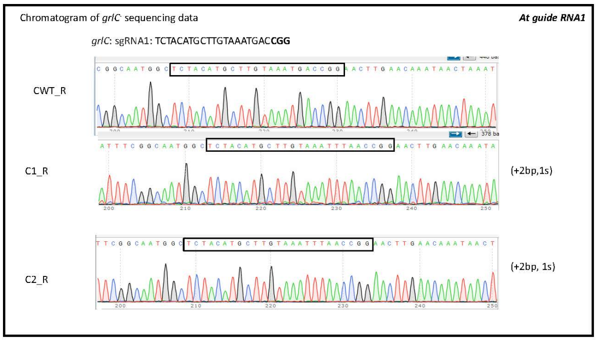

1

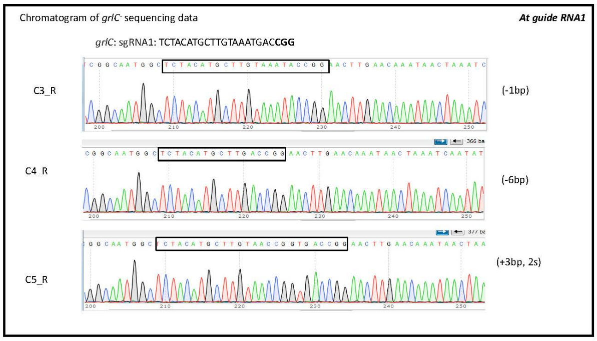

2

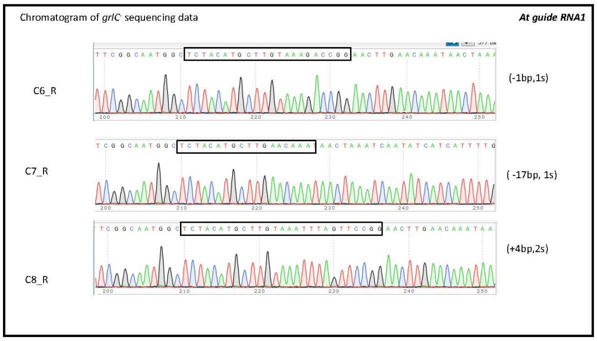

3

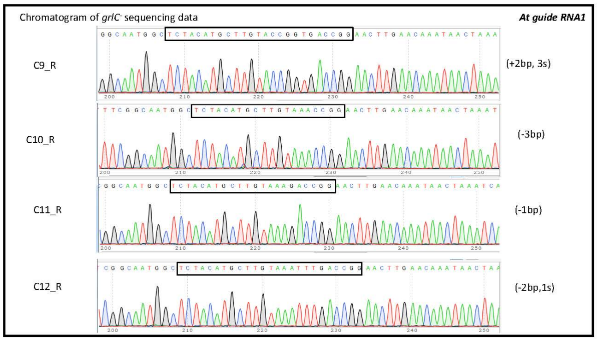

4

grlC sgRNA2

S. Fig. 4a

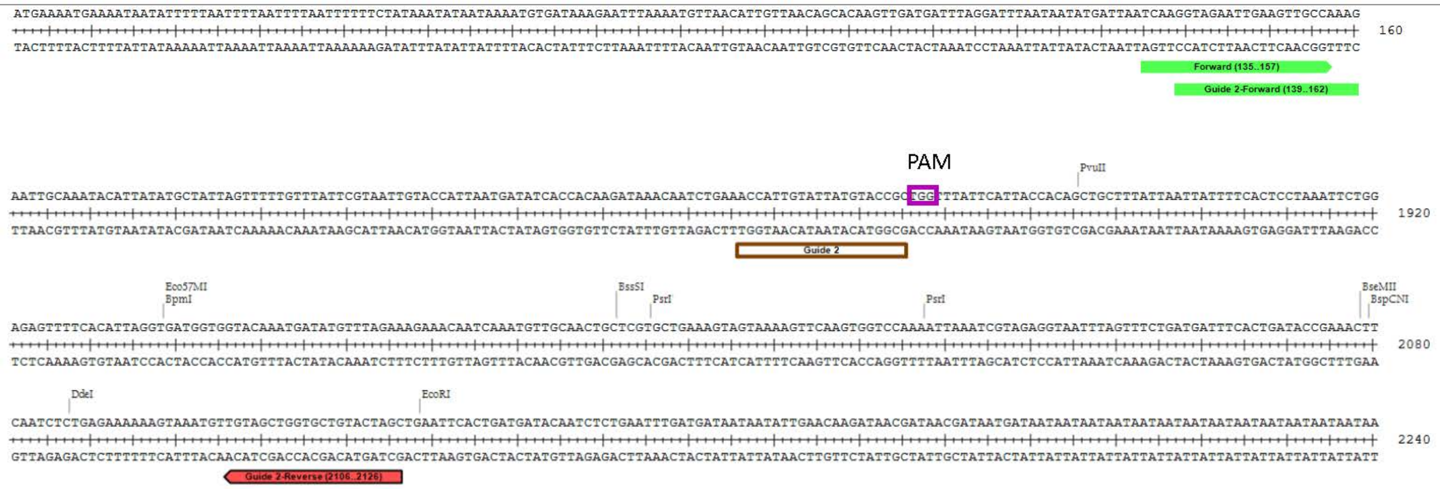

Guide2
F: 5'-GGTAGAATTGAAGTTGCCAAAGGT(139bp - 162bp)
R: 5'-GCTAGTACAGCACCAGCTACA(2106bp - 2126bp)
Product size:1987bp

Reversed primer was used for sequencing

6/27/2020

S. Fig.4b

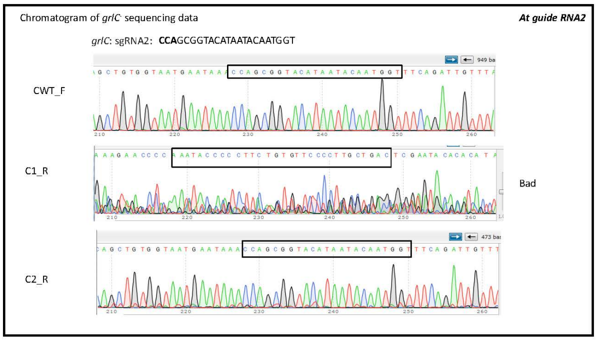

1

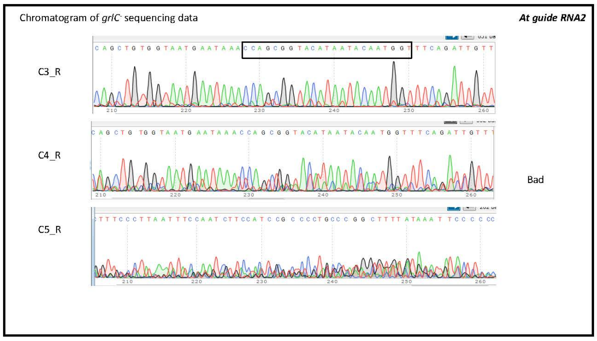

2

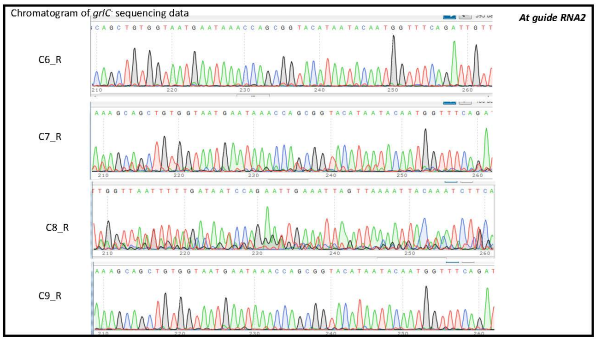

3

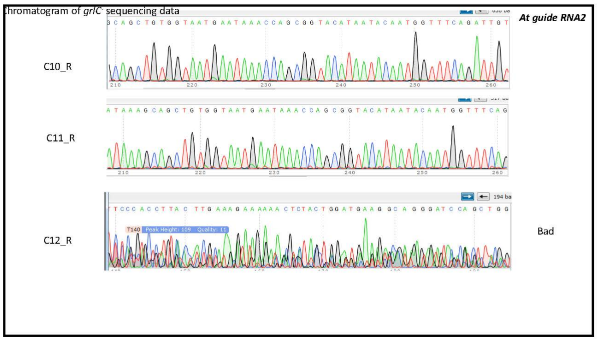

4

S. Fig. 5

Dync1li1

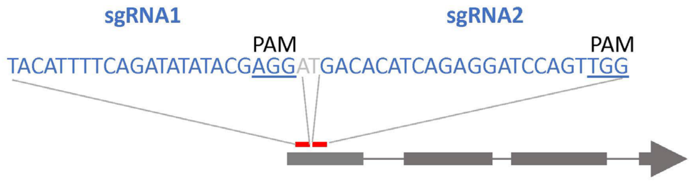

DNA

```
WT  ATACATTTTCAGATATAT-----ACGAGGATGACACATCAGAGGATCCAGTTGGTAGAATC
1   ATACATTTTCAGATATAT-----ACAAGGATGACACATCAGAGGATCCAGTTGGTAGAATC 1s
2   ATACATTTTCAGATATAT-----ACGAGGATGACCCATCAGAGGATCCAGTTGGTAGAATC 1s
3   ATACATTTTCAGATATATCGTACGAGGATGACACATCAGAGGATCCAGTTGGTAGAATC +3bp
4   ATACATTTT------------------------------------------CATCAGAGGATCCAGTTGGTAGAATC -21bp
```

Protein

```
WT   I A L S Y T F S D I - Y E D D T S E D P V G R I N
N1   I A L S Y T F S D I - Y K D D T S E D P V G R I N
N2   I A L S Y T F S D I - Y E D D P S E D P V G R I N
N3   I A L S Y T F S D I S Y E D D T S E D P V G R I N
N4   I A L S Y T F S - - - - - - - - S E D P V G R I N
```

DNA

```
WT  ATACATTTTCAGATATATACGAGGATGACACATCAGAGGATCCAGTTGGTAGAATC
1   ATACATTTTCAGATATATACAAGGATGACACATCAGAGGATCCAGTTGGTAGAATC 1s
2   ATACATTTTCAGATATATACGAGGATGACACATCAGAGGATCCTGTTGGTAGAATC 1s
3   ATACATTTTCAGATATATACGAGGATGACACATCAGAG----------GTTGGTAGAATC -6bp
4   ATACATTTTCAGATATATACGAGGATGACACATCA----------------GTTGGTAGAATC -9bp
5   ATACATTTTCAGATATATACGAGGATGACACATCAGAGGATC-----TTGGTAGAATC -3bp
6   ATACATTTTCAGATATATACGAGGATGACACATCAGAGGA----AGTTGGTAGAATC -3bp
7   ATACATTTTCAGATATATACGAGGATGACACATCAGAGGAT-----GTTGGTAGAATC -3bp
```

Protein

```
WT   T F S D I Y E D D T S E D P V G R I N Y W S L E G
N1   T F S D I Y E D D T S K D P V G R I N Y W S L E G
N2   T F S D I Y E D D T S E E R V G R I N Y W S L E G
N3   T F S D I Y E D D T S - - E V G R I N Y W S L E G
N4   T F S D I Y E D D T - - - S V G R I N Y W S L E G
N5   T F S D I Y E D D T S E - D L G R I N Y W S L E G
N6   T F S D I Y E D D T S E - E V G R I N Y W S L E G
N7   T F S D I Y E D D T S E - D V G R I N Y W S L E G
```
